# High Stability and Low Power Nanometric Bio-Objects Trapping through Dielectric–Plasmonic Hybrid Nanobowtie

**DOI:** 10.3390/bios14080390

**Published:** 2024-08-13

**Authors:** Paola Colapietro, Giuseppe Brunetti, Annarita di Toma, Francesco Ferrara, Maria Serena Chiriacò, Caterina Ciminelli

**Affiliations:** 1 Optoelectronics Laboratory, Politecnico di Bari, Via E. Orabona 6, 70125 Bari, Italy; p.colapietro@phd.poliba.it (P.C.);; 2 CNR NANOTEC-Institute of Nanotechnology, Via per Monteroni, 73200 Lecce, Italy; francesco.ferrara@nanotec.cnr.it (F.F.);

**Keywords:** optical trapping, virus, nanomanipulation

## Abstract

Micro and nano-scale manipulation of living matter is crucial in biomedical applications for diagnostics and pharmaceuticals, facilitating disease study, drug assessment, and biomarker identification. Despite advancements, trapping biological nanoparticles remains challenging. Nanotweezer-based strategies, including dielectric and plasmonic configurations, show promise due to their efficiency and stability, minimizing damage without direct contact. Our study uniquely proposes an inverted hybrid dielectric–plasmonic nanobowtie designed to overcome the primary limitations of existing dielectric–plasmonic systems, such as high costs and manufacturing complexity. This novel configuration offers significant advantages for the stable and long-term trapping of biological objects, including strong energy confinement with reduced thermal effects. The metal’s efficient light reflection capability results in a significant increase in energy field confinement (EC) within the trapping site, achieving an enhancement of over 90% compared to the value obtained with the dielectric nanobowtie. Numerical simulations confirm the successful trapping of 100 nm viruses, demonstrating a trapping stability greater than 10 and a stiffness of 2.203 fN/nm. This configuration ensures optical forces of approximately 2.96 fN with an input power density of 10 mW/μm^2^ while preserving the temperature, chemical–biological properties, and shape of the biological sample.

## 1. Introduction

The research field on nanoparticle optical manipulation is going through a period of great dynamism, motivated by the growing need for precise control over the translation and rotational dynamics of nanoparticles for diagnostic, therapeutic, or research purposes. The use of optical techniques for the non-contact handling/manipulation of nanoparticles represents an innovative approach to achieving these goals, as it facilitates studies that control the individual or collective behavior of particles under the influence of light. In this context, the availability of flexible and multifunctional optical tweezers perfectly meets the demand to develop and use new methods for efficiently and precisely trapping or controlling biological objects at the micro/nanometric scale. Therefore, these tools have significantly impacted scientific and engineering research, opening new perspectives into manipulating biological particles at previously unexplored dimensional scales. This suite of new techniques overcomes the constraints of conventional approaches, offering novel opportunities to engineer solutions that could revolutionize the modalities of treatment and follow-up of diseases, spanning from oncological, neurodegenerative, or infective diseases and deep biological understanding for pharmaceutical and diagnostic purposes.

The first study and demonstration of optical trapping in biology for dielectric particles by a single-beam gradient force trap appeared in 1986 by A. Ashkin, when an individual tobacco mosaic virus [1], an Escherichia Coli bacterium [1], and a live single cell [2] were trapped. Optical tweezers use the force exerted by a strongly focused beam of light to trap with ultra accuracy and dynamically move micro/nano-sized objects. Ashkin’s original design has been adapted for a variety of purposes, such as trapping and manipulation/inactivation of biological materials in the fields of physics, nanoscience, biology, and life sciences, contributing to advancements in personalized and modern medicine science, such as monitoring inter- and intracellular processes or the manipulation of viruses and bacteria [3]. Indeed, optical trapping configurations have been developed to trap small cells and microorganisms [4], first preventing contact between the target and the device and then damaging or disrupting the target object, as required in non-invasive medicine procedures.

However, as widely discussed in the literature [5,6], optical tweezer nanocavities encounter several limitations in interacting with biological nanoparticles, such as the diffraction limit of the focused trapping laser spot size and thermal challenges. While these factors hinder accurate trap confinement [7], on the other hand, they induce undesirable local heating of the biological sample under test. The overheating could lead to structural alterations, compromising biological properties and functions, biological membranes, and even denaturing the trapped particle. This phenomenon is particularly pronounced in the case of viruses, as numerous studies have shown that their protein envelope is highly dynamic and sensitive to significant temperature variations [8].

The above limitations make trapping and manipulating micro- and nano-sized particles particularly challenging, which is why the scientific community has investigated several new approaches and configurations that could solve the issues by enhancing the trapping capacity by exploiting evanescent fields.

Photonic crystals (PhCs) represent a promising solution in supporting these new approaches. PhCs are near-field nanostructures characterized by a periodic pattern in dielectric properties that have already demonstrated the efficient trapping of single proteins by exploiting a power of 10 mW to achieve a stiffness of the order of hundreds of fN/nm [9].

Moreover, in recent years, nanobowties have attracted increasing attention due to their capacity to address the previously mentioned limitations. Among the various strategies and configurations for nanoscale trapping, nanobowties represent the most promising geometry for high-precision optical tweezer applications, owing to their distinctive non-invasive properties and high electric field enhancement [10]. For instance, ref. [11] reports on the numerical study of the optical forces generated by a nanobowtie antenna, demonstrating that very high values can be produced when the incident light polarization is along the bowtie gap. However, it has been shown that the optical trapping efficiency of nanobowtie can exceed the performance of conventional numerical aperture optical traps by approximately 20 times [12].

Nanobowties are a promising and versatile choice for advanced biological trapping applications due to their ability to concentrate the electric field very efficiently, their compatibility with existing fabrication techniques, and their easy integration into more complex structures.

Plasmonic nanostructures are the most used because of their strong energy confinement, demonstrating the trapping of single proteins, DNA/RNA for subsequent analysis [13], or 3D manipulation with tweezers placed on an optical fiber tip [14]. However, the main issue with plasmonic configuration is the higher thermal effect, which could damage the bio-object under test.

Dielectric configurations, such as ring resonators and dielectric nanoantennas, have also been exploited as optical nanotweezers [15,16] because they overcome the thermal challenge even if their energy confinement is weaker than the plasmonic ones.

Therefore, the hybrid dielectric/plasmonic cavities represent a better compromise to exploit the advantages of both plasmonic and dielectric configurations: strong energy confinement with lower thermal effects. For example, in ref. [17], the Authors designed a photonic/plasmonic device with a 1D PhC dielectric cavity vertically coupled to a metal bowtie to trap a single 200 nm Au bead with P_in_ = 190 μW. This configuration allows high Q-factors and low mode volumes, overcoming the diffraction limits and avoiding direct contact with the matter to be trapped. However, vertical coupling requires a precise alignment between the PhC and the metal structure because any misalignment can significantly reduce the light coupling efficiency and the trapping performance. Moreover, the fabrication of such structures is significantly complicated and costly.

To overcome these limits, we propose, for the first time in the literature to our knowledge, a design of an inverted hybrid nanobowtie. The device, based on silicon-on-insulator technology, consists of two tip-to-tip triangular silicon elements (TTSWs) with a metal layer positioned beneath them and the underlying silicon oxide substrate designed for trapping beads. The metal layer can effectively reflect light, creating an electric field confined to the region near the metal surface. The triangular shape of the TTSWs guides the reflected light toward the central area between the two tips. As a result, the electromagnetic energy confined in the desired site, namely on the tips of TTSWs, is increased with a consequent high value of the trapping stability (>10), as demonstrated by numerical simulations.

These features, combined with the robustness of fabrication, make the proposed device highly promising for the stable and long-term trapping of nanoparticles and multifunctional applications based on light–matter interactions. As a future perspective, a hybrid dielectric–plasmonic nanobowtie array could be used for multiple trapping followed by plasmonic staining, fluorescence, and SERS imaging.

**Figure 1:**
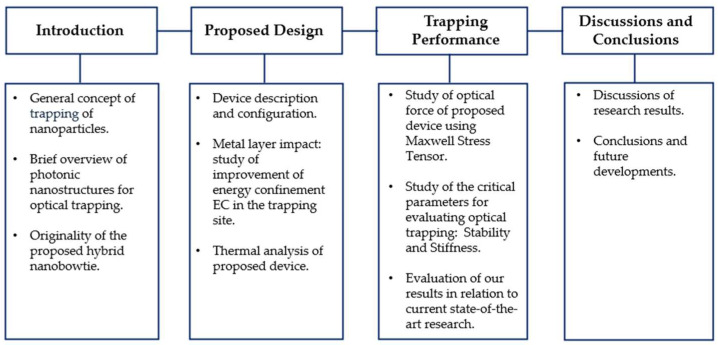
Schematic workflow diagram of the research article.

The current Section provides an overview of the main optical nanocavities for trapping micro and nano biological objects and the motivations for the investigation of the inverted hybrid nanobowtie, while Section 2 will detail the proposed device’s design, discussing the results obtained from FEM simulations of its electromagnetic and thermal behavior. In Section 3, the trapping performance of the proposed device will be evaluated, comparing the obtained results with the current state-of-the-art. Finally, Section 4 will discuss the research results, concluding with a comprehensive summary of the study. Figure 1 shows a block diagram that outlines the work presented in the following article.

## 2. Proposed Design

**Figure 2:**
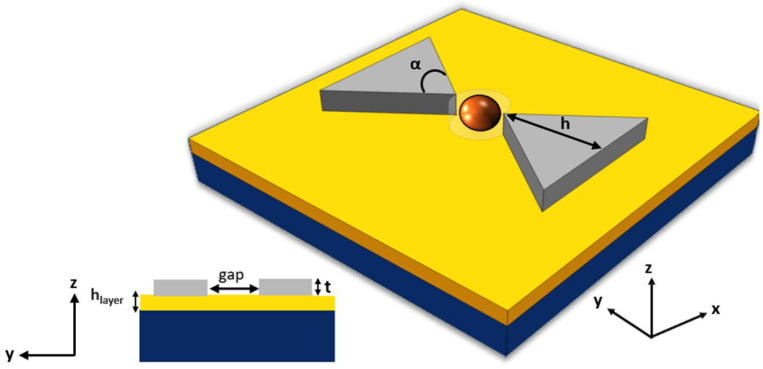
Schematic of the investigated nanobowtie in SOI platform with a metal layer.

The proposed device is a silicon-on-insulator (SOI) nanocavity comprised of two silicon TTSWs with a metal layer, forming the nanobowtie structure illustrated in Figure 2. In this configuration, the selected metals are silver and gold. The thickness, h_layer_, of the metal layer underneath the two TTSWs has been dimensioned, for both metals, aiming at optimizing the energy confinement in the trapping site.

The two TTSWs have a thickness (t) of 220 nm, a height (h) of 260 nm, and an angle (α) of 110° [16]. They are positioned above a silicon oxide (SiO_2_) layer, separated by a gap region that serves as the trapping site. This area is modeled as a dotted cylinder with a diameter gap of 120 nm, which is the optimal size to prevent particle adhesion to the tips.

The SiO_2_ substrate is modeled with a thickness on the order of millimeters. Specifically, it is approximately four orders of magnitude thicker than the trapping site, allowing the substrate to be infinitely extended by employing perfectly matched layers (PMLs) in COMSOL Multiphysics^®^. The PML establishes a perfectly absorbing domain as an alternative to non-reflecting boundary conditions.

The origin of the xyz coordinate system coincides with the center of the base of the cylinder at the dielectric/metal interface. The two TTSWs are positioned symmetrically with respect to the center of the trapping site, with a plane wave out-of-plane normal light excitation along the opposite direction of the z-axis and polarization along the y-axis to excite the so-called “slot” effect that ensures the confinement of the electromagnetic energy density within the trapping site.

A diameter of 100 nm was assumed for the trapped particle. This parameter is a key point in the scale of biological entities since it could correspond to the typical diameter of a virus, such as those belongings to the Coronaviridae or Paramyxoviridiae virus family, Influenza A or HIV [18,19], as well as to small extracellular vesicles, namely exosomes. These exosomes are membrane-enclosed particles physiologically produced by all the cells in organisms, mainly for intercellular communications, and are increasingly recognized as potential biomarkers of various pathologies [20].

The proposed device is surrounded by an aqueous medium needed to model the reaction and transport of multiple biospecies. We assume an operating wavelength λ_0_ of 1550 nm. The advantage of shorter wavelengths in reducing particle scattering force is countered by the proportional increase in gradient force with wavelength. Therefore, higher wavelengths result in a stronger gradient force, enabling more precise and controlled particle trapping [4]. Additionally, using lasers at telecommunication wavelengths offers significant flexibility in both polarization and power. Moreover, the moderate value of the water absorption coefficient at 1550 nm confirms its suitability for biological trapping applications, as also demonstrated in ref. [15,21,22,23].

The refractive index @1550 nm for Si, SiO_2_, H_2_O, Au, and Ag are n_Si_ = 3.48, n_SiO2_ = 1.444, n_H20_ = 1.318 + 9.86 × 10^−5^i, n_Au_ = 0.52406 + 10.742i, and n_Ag_ = 0.40960 + 10.048i, respectively [24,25,26,27,28].

For spherical particles of 100 nm diameter, a refractive index of 1.50 has been assumed, consistent with what has been experimentally demonstrated in the characterization of viruses, such as Influenza A [29,30] and HIV-1 [31].

To ensure that the maximum electromagnetic energy density is confined in the trapping site, the continuity condition of the normal component of the electric field displacement has been imposed. This boundary condition ensures the conservation of electromagnetic energy across the dielectric–metal interface, characterized by a high index contrast. It promotes a substantial confinement of light and an increased electric field near the TTSW-shaped tips of each TTSW.

The energy field confinement within the trapping site has been maximized by engineering, and the device structure has been optimized by adding a metal layer. The improvement achieved with this solution is highlighted by comparing it to the same structure without the layer.

Energy confinement (EC) at the trapping site has been assumed to be the figure of merit addressing the requirement for effective energy confinement within the trap to ensure strong trapping capabilities even with a compact volume. It is defined as the ratio between the integral of the electromagnetic energy density in the trapping site and the one in the whole volume surrounding the nanocavity, as described by the equation in ref. [16].

**Figure 3:**
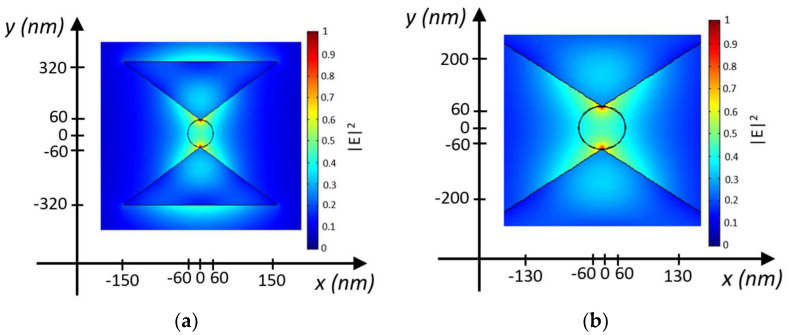
(**a**) Electric field intensity distribution at the trapping site in the middle plane of the nanostructure at z = 200 nm + t_Ag_ for the bowtie with t_Ag_ = 50 nm by considering P_in_ = 10 mW/μm^2^. (**b**) Zoom-in of (**a**).

The electric field intensity distribution in the middle plane of the nanostructure at the trapping site for the proposed configuration is shown in Figure 3, with an input optical power P_in_ = 10 mW/μm^2^, calculated using the finite element method (FEM) approach, by assuming a silver layer 50 nm thick. The predominant orientation of the electric field occurs along the z-direction, indicating the quasi-TE polarized mode in the nanocavity. The electromagnetic energy density is confined within the gap region, with minimal diffusion into the bowtie.

**Figure 4:**
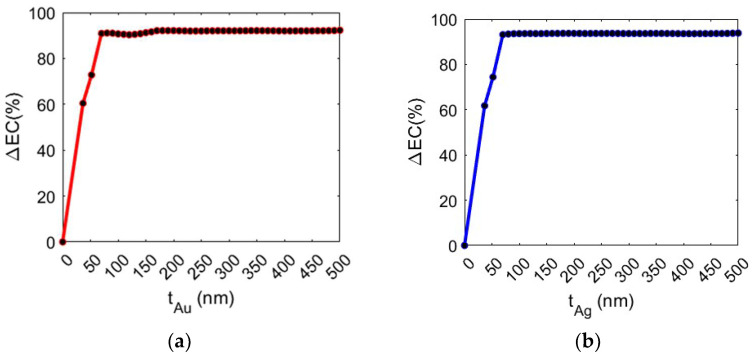
(**a**) ΔEC (%) vs. metal layer thickness (nm) with gold and (**b**) silver layer.

Analyses were carried out by considering both a gold and silver layer. The study was conducted by varying the thickness (t) of the metal layer, evaluating the increase in the percentage of EC (ΔEC) in the volume of the trapping site in comparison to the values obtained without the metal layer (EC = 3.75 μV/m). As shown in Figure 4, ΔEC increases up to 70 nm with the gold layer and 50 nm with the silver layer and subsequently saturates. Specifically, the configurations with t_Au_ = 70 nm and t_Ag_ = 50 nm show an EC increase of 91.93% and 92.20%, respectively.

The presence of a metal layer results in a significant increase in the electric field confinement (EC), enhancing light–matter interactions and improving the efficiency of the nanocavity for optical trapping applications involving biological objects.

The trend saturates for thickness values of the Ag and Au layers, respectively equal to 50 nm and 70 nm, due to the presence of energy losses or interference phenomena that can further limit the effectiveness of increasing the metal thickness beyond these values.

When designing a device for optical trapping, it is crucial to manage and regulate thermal effects to prevent potential overheating that could compromise the behavior and biological functions of the trapped nanoparticles, ensuring unreliable and inconsistent results. This is supported by studies demonstrating the highly dynamic and temperature-sensitive nature of the viral envelope, confirming that many viruses, including SARS-CoV-2, are easily deformable [8]. Overall, overcoming the challenges posed by heating within nanocavities is a key goal in designing high-performing structures for nanoparticle trapping in several scientific and technological domains.

Thermal behavior has been numerically simulated through 3D COMSOL Multiphysics^®^ Software version 4.4 by combining heat transfer and optical physics. The value of the energy dissipated in the nanoparticle volume was initially determined before calculating the temperature variation at the trapping site. Indeed, the temperature equation defined in solid domains corresponds to the differential form of Fourier’s law, allowing the calculation of the temperature variation value at a point in the trapping site.

**Figure 5:**
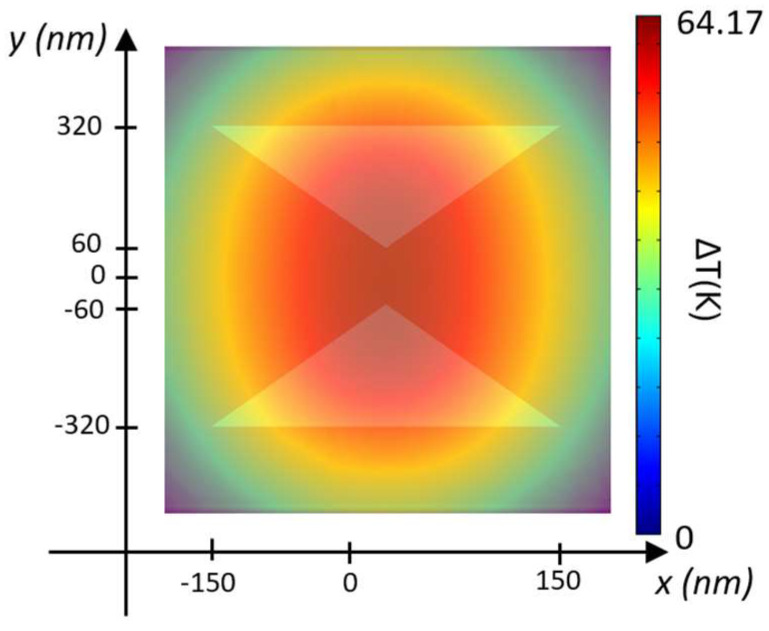
Steady-state temperature rise distributions ∆T(K) = T − T_0_, T_0_ = 293.15 K for the silver layer configuration at z = 200 nm + t_Ag_ for P_in_ = 10 mW/μm^2^.

Figure 5 provides a top-down view of the steady-state temperature distribution for the silver layer configuration with t_Ag_ = 50 nm, assuming an input optical power of 1 mW/µm^2^.

As illustrated in Figure 5, temperature increases occur at the gap between the two silicon TTSWs, specifically in the trapping site. In the configuration with the gold layer (t_Au_ = 70 nm), the temperature rises by ∆T = 6.52 K at P_in_ = 1 mW/μm^2^, reaching ∆T = 64.17 K at P_in_ = 10 mW/μm^2^. Similarly, with the silver layer (t_Ag_ = 50 nm), the temperature increase is ∆T = 6.39 K at P_in_ = 1 mW/μm^2^ and ∆T = 63 K at P_in_ = 10 mW/μm^2^. Thus, increasing the input power density results in a substantial temperature rise in both configurations, even with a slightly greater increase in the case of the gold layer. These results are promising as they allow for working with temperature values that preserve the vitality of the trapped biological species without causing any thermal damage or inactivation. This confirms the potential of the proposed hybrid nanobowtie cavity as a robust and high-performance trapping device in the biological field.

**Figure 6:**
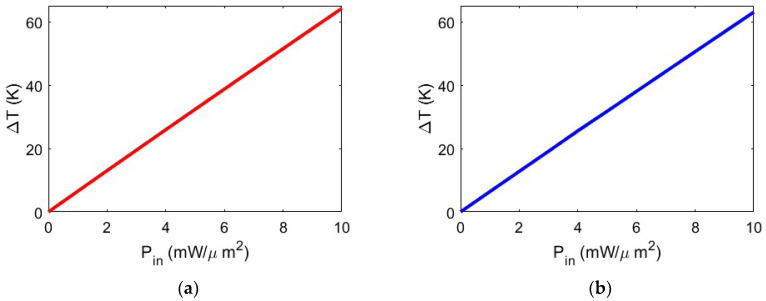
(**a**) Temperature increase ∆T in the trapping area as a function of the input optical power for nanocavity with gold layer; (**b**) silver layer.

In this regard, a relevant and interesting study was conducted in ref. [32] by analyzing inactivation temperature values of SARS-CoV-2, which vary depending on viral strains and culture media. A reasonable estimate suggests that temperatures exceeding 65 °C will result in almost complete inactivation for any viral strain with exposures lasting more than 3 min. Considering this, a crucial aspect to be considered during the design of nanocavities for virus trapping is the selection of input power density values that determine the heating temperature within the trapping site. In Figure 6, we show the temperature at the trapping site as a function of trapping laser power density intensity, varying from P_in_ = 1 mW/μm^2^ to P_in_ = 10 mW/μm^2^ for both configurations. Figure 6 clearly shows a linear dependency between input power and temperature variation within the trapping site. The values shown provide precise and stable trapping and, being below threshold values, ensure that the nanoparticle undergoing heating remains intact, thus preserving its native characteristics and properties. In this manner, it is possible to use the proposed configuration for pharmaceutical and/or diagnostic purposes.

## 3. Trapping Performance

In this study, we performed three-dimensional finite element method (FEM) simulations using COMSOL Multiphysics^®^ Software to analyze the distribution of near-field, optical force, potential energy, and temperature rise [33]. The simulations were carried out using the Electromagnetic Waves, Frequency Domain module in COMSOL Multiphysics^®^, used to solve for the time–harmonic electromagnetic field distributions. Perfectly matched layers (PMLs) have been applied at the top and bottom of the computational domain to absorb the scattering light in the far field. The boundary condition at the interfaces was treated as a continuous one. All blocks of the geometry were divided into many small tetrahedron meshes for the 3D areas and triangle meshes for boundaries, the smallest length of which could reach 10 nm. A Helmholtz equation was built on the meshed geometry to describe the near-field scattering process and discretized at every mesh point to form a large sparse matrix. Finally, the numerical solutions of the Helmholtz equation were obtained by solving the built matrix using FEM.

Following the method outlined in ref. [15,34], we assessed the Maxwell stress tensor (MST) to calculate the optical forces exerted on particles suspended in the electromagnetic field. The optical force is generated by momentum transfer due to the interaction between the incident electromagnetic field and dielectric particles. Specifically, we calculated the optical force by computing the surface integral over a sphere with a diameter 10 nm larger than the particle itself. Corresponding to the refractive index of the virus, the sphere with the largest diameter also shows a refractive index of 1.50.

This integral involved the vector product of the Maxwell stress tensor (MST) and the outward vector normal to the surface. The optical force can be expressed as:

(1) $$F=\oint_{s}^{\;}\left(T_{M}\times n\right)dS$$

where T_M_ is the Maxwell stress tensor, S is the particle surface, and n is the outgoing vector normal to the surface. The force is integrated in one dimension to obtain the trapping potential energy under the simplifying assumption, which is a conservative force. Overall, the Maxwell stress tensor is often used in a trapping context to evaluate the resultant force on particles, allowing a detailed understanding of the dynamics of optical trapping.

**Figure 7:**
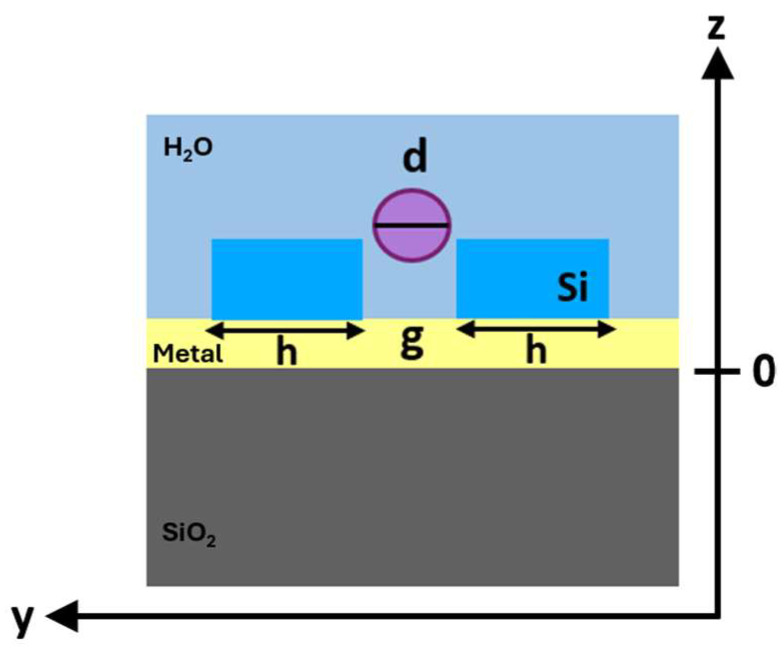
Schematic and lateral view of the nanocavity (z–y directions) with a trapped particle (d = 100 nm).

Therefore, to estimate the trapping efficiency at 1550 nm of both proposed configurations and confirm the benefits related to the insertion of the metal layer, the optical force on the target particle was calculated by varying the position of the bead along the z-axis. The schematic and lateral view (z–y directions) of the investigated nanobowtie configuration with a trapped particle is shown in Figure 7.

Researchers have studied different optical dimensional regimes based on the size of the target to be trapped to investigate the behavior of the optical forces. In this work, the Rayleigh regime has been considered [4], as it is best suited for trapping particles smaller than the wavelength [35], providing an accurate basis for studying light–matter interactions in biological and medical applications involving nanometric objects, such as viruses.

**Figure 8:**
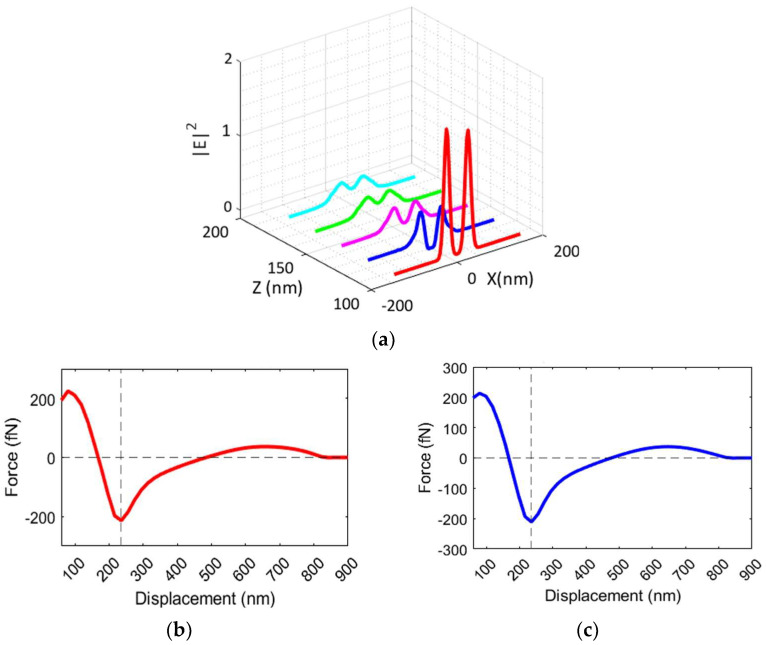
(**a**) Representation of the intensity of electric field along the trapping site of the nanocavity. (**b**) Optical force trend vs. displacement of nanoparticle in configuration with gold layer (**c**) and silver layer.

Figure 8a shows the intensity of the electric field along the trapping site of the nanocavity, modeled as a cylinder in the direction of the particle movement (along the z-axis). In line with what is shown in Figure 3, the electric field strength is maximum at the tips of the two silicon TTSWs and decays with a standard deviation σ of 6.2789 in the regions outside the trapping site until it reaches zero. The parameter σ represents the decay coefficient of the electric field distribution, which is roughly approximated by a Gaussian distribution.

This gradual decay is characteristic of an evanescent field, which plays a crucial role in the trapping mechanism by providing a localized high-intensity field region that traps particles within the nanocavity, exerting a strong trapping force without the need to increase the incident power density.

The analysis examines the behavior of the electric field at five different positions along the cylinder: the red curve represents the electric field intensity for z = 100 nm, where the particle is trapped and the intensity is at its maximum. As the distance increases for z > 100 nm, the electric field intensity tends to decrease, as shown by the blue, magenta, green, and cyan curves, which follow the optical force trends illustrated in Figure 8b,c. Indeed, Figure 8b,c shows the vertical force F_z_ on the sphere as a function of its position with P_in_ = 10 mW/μm^2^. The optical force trends are very similar in both configurations: more specifically, for z < 170 nm, the particle tends to rise upwards by a positive F_z_ optical force, while for z > 170 nm, the F_z_ is negative, guiding the particle downwards. The peak of the optical force F_zmax_ = 2.96 fN occurs at z_max_ = 80 nm for a nanobowtie with a gold layer and F_zmax_ = 2.14 fN in z_max_ = 80 nm for a nanobowtie with a silver layer.

When a particle is flowing in a region near the metal layer where optical forces are significant, it is primarily influenced by an attractive positive force guiding it around its equilibrium point (x = 0, y = 0, z = 80 nm), where attractive and repulsive forces balance and the field gradients are the largest, enabling stable trapping. The positive optical force is associated with an increase in the value of the electric field at the specific point. Afterwards, the particle undergoes a negative optical force, which corresponds to a reduction in field strength and tends to decrease. For outer values, the particle can be considered free to move.

Through optical force calculations, two additional critical parameters for evaluating optical trapping can be extracted: stability and stiffness. Two ways to improve these trapping parameters are the enhancement at the trapping site and the strong field confinement. The trapping stability in an optical cavity is defined as the ratio of potential energy to thermal energy and refers to the capability to maintain a particle in a stable and controlled position within the generated optical field.

More specifically, an effective and stable trap requires that the optical forces are greater than the repulsive thermal force, which might otherwise push the particle away from the equilibrium point. A high value of the trapping stability makes the proposed device very promising for long-time particle immobilization.

The stability of the nanocavity has been estimated as

(2) $$S=\frac{U}{K_{b}T_{c}}$$

where *U* [J] is the potential energy corresponding to the work required to bring the nanoparticle from a free position to the trapping site. *U* can be expressed as $U\left(r\right)=\int_{\infty }^{r_{0}}F\left(r\right)\cdot dr$, with *F*(*r*) denoting the optical force exerted on the particle moving along the cylinder representing the trapping site. *K_b_* is the Boltzmann constant [J/K], and *T_c_* is the temperature in Kelvin [K], also considering the temperature rise ∆T due to the trapping effect [33].

**Figure 9:**
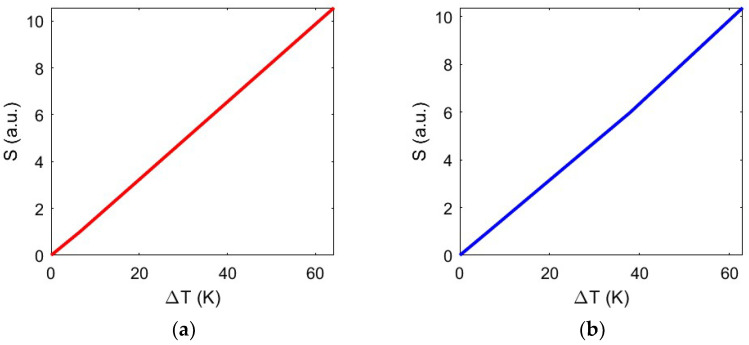
(**a**) Stability trapping vs. ∆T for nanobowtie with gold layer; (**b**) for nanobowtie with silver layer.

In a stable trap, despite small perturbations and mechanical fluctuations, the particle tends to remain in its equilibrium position. However, the stability can be impacted by several factors, including the trap’s geometric configuration, the size and the shape of the object to be trapped, the characteristics of the forces involved, and, most importantly, the temperature. In this regard, Figure 9 illustrates the study of the stability concerning the temperature variation reached at the trapping site, demonstrating a linear dependence between the two quantities.

Therefore, Kramers’ theory [36] states that the trapping time (t_trap_) is inversely proportional to the stability of the potential depth. Increased stability, therefore, corresponds to a longer trapping time, which can be affected by various factors, including temperature, mechanical vibration, acoustic noise, as well as fluctuations in the laser input power. In particular, t_trap_ ≈ exp[U/(K_b_T)] and, hence, the potential energy should be greater than or equal to K_b_T. For successful trapping to take place, the trapping stability value must exceed one. When stability numbers fall below the unit, the trapping is regarded as weaker than the local Brownian motion of the particle, making successful trapping highly unlikely in such cases. Therefore, stability values slightly greater than 1 are necessary to ensure the dominance of optical forces over thermal forces but are not sufficient to trap the target for an extended period. Indeed, trapping times of only several seconds have been observed with other dielectric nanotweezers having S~1 [15,16,36].

A more accurate approach for studying and evaluating the trapping stability of optical nanocavity requires the potential depth to be greater than 10 K_b_T to relieve the Brownian motion of particles caused by thermal perturbations [35,37,38]. The stability of the nanoparticle trap is temperature sensitive, confirming that the dependence of the Brownian motion velocity on temperature plays a crucial role in the release of trapped nanoparticles. For this reason, it is essential to optimize the trapping forces. Therefore, considering the disturbances induced by instantaneous Brownian effects, the trapping potential must be significantly greater than the thermal energy; the ratio of trap energy to thermal energy should be 10 or higher for the nanoparticles to remain in the trap.

A stability of 10 means that the optical forces are 10 times stronger than the repulsive thermal forces, allowing the optical trap to persist for extended periods without losing effectiveness. However, the studies reported in ref. [39,40,41,42] have employed the criterion of S = U/(K_b_T_c_)~10, demonstrating that this method is highly effective in quantitatively describing trapping stability. Also, in ref. [43], they imposed S = U/(K_b_T_c_) ≥ 10 as a threshold criterion and demonstrated the trapping of 26 nm diameter silica beads with a laser power of 1.5 W. Since the input optical power is a necessary control parameter to overcome Brownian forces and to modify the trapping stability value, we carried out studies by varying the input power P_in_ up to 10 mW, such as in ref. [43]. The aim was to obtain a trapping stability value of 10 or slightly higher while ensuring that the temperature increase resulting from this increase does not induce virus denaturation.

An input power of 10 mW can, therefore, generate more intense optical forces, thereby increasing trapping stability. This is particularly useful for nanoparticles such as viruses or small vesicles, where much higher forces are required for effective trapping over long periods. This is because, as the particles become smaller, their polarizability decreases, necessitating an increase in laser intensity. Thus, the intensity of the incoming laser beam, understood as the focused power density, is chosen according to the radius of the particle, especially when it is smaller than the wavelength of light. For example, a 10 mW laser focused into a 1 μm^2^ area would only trap particles with a radius of 56 nm or more. Meanwhile, a 100 mW laser would only trap particles with a radius greater than 25 nm [44].

The increased input power density elevates the temperature in the trapping volume, providing the device with greater resistance to external perturbations, such as mechanical vibrations. This contributes to enhancing the robustness and stability of the optical trapping nanocavity. Higher stability also enhances control over the optical trap, allowing for more precise confinement of the nanoparticle. This is particularly crucial in specific applications requiring good reproducibility and when trapping particles for a prolonged time is necessary. However, it is crucial to consider possible disadvantages or side effects associated with higher power density, such as the risk of overheating biological matter and the subsequent probability of related damage. For instance, trapped latex spheres of 109 nm in diameter were destroyed by a 15 mW beam in 25 s, which has serious implications for biological matter [3]. Indeed, in the design criteria of trapping nanocavity, a compromise must be reached by carefully balancing the input power density with the need to avoid overheating and damage to biological matter.

In this work, with a power density of P_in_ = 10 mW/µm^2^, a temperature variation at the trapping site of 63 K and 64.17 K is achieved with, respectively, the silver layer and the gold layer, aiming at optimal performance in terms of stability while simultaneously falling within critical temperature limits and working with not too high input optical power values. Indeed, ref. [45] demonstrated stable trapping with S = 10 of a 75 nm nanoparticle for an input power of 250 mW. Considering an input power density of 10 mW/µm^2^, a stability trapping of 10.370 was obtained for the nanobowtie configuration with the silver layer and 10.565 for the nanobowtie configuration with the gold layer, more than 10x with respect to the value reported in refs. [15,16,37].

Below is a summary table (Table 1) comparing the stability trapping values obtained in this study (points 5 and 6) to the input power used and the size of the trapped particles with other existing state-of-the-art values.

**Figure 10:**
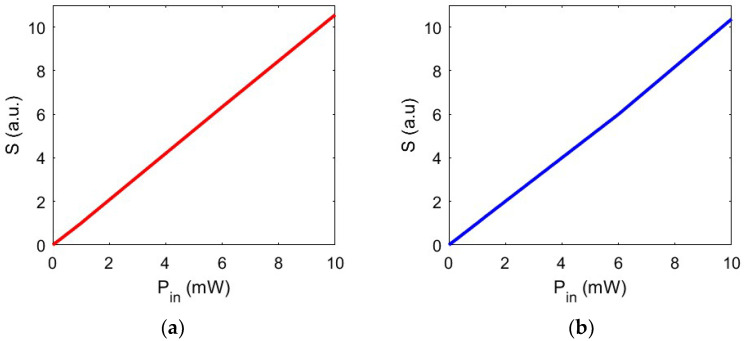
(**a**) Stability trapping by varying the input power P_in_ for nanobowtie with a gold layer; (**b**) for nanobowtie with a silver layer.

Figure 10 shows the trapping stability of the proposed configurations as a function of the input power P_in_. The analysis reveals the limiting value associated with the input power density and, consequently, the temperature change reached at the trapping site. Below this threshold, the stability decreases to a value below unity, rendering the device less promising for trapping nanoparticles for extended periods. With P_in_ < 0.7 mW/μm^2^, S < 1.

The stability results obtained for both nanobowtie configurations, which also indicate a prolonged trapping time for the nanoparticle, show a significant improvement in this parameter compared to other configurations previously reported in the literature, specifically in comparison to ref. [15]. While ref. [15] achieves a stability of 1 with an input power of 6 mW/μm^2^, the configuration proposed here achieves the same stability value with an input power of only 1 mW/μm^2^. This improvement can be attributed to the increased confinement of the electric field within the trapping site, with high values near the tips of the two silicon tip-to-tip elements and a consequent significant increase in the light–nanoparticle interactions.

The behavior of optical forces and the stability of trapping in optical cavities are significantly influenced by the size of the trapped particles. The trapping of particles with a diameter (d) smaller than the wavelength (d < λ) is more challenging than trapping microscale objects, as the optical forces are proportional to the cube of the particle radius, which varies from a few nanometers to several hundred nanometers. Optical forces are typically decomposed into two components: a scattering force in the direction of light propagation and a gradient force in the direction of spatial light. The stability of the trapping depends on the balance between these forces. A larger particle diameter results in a stronger scattering force, which reduces trapping stability. The scattering force tends to move the particle in the direction of light propagation, opposing the gradient force that seeks to trap the particle at the point of maximum electromagnetic field strength. When the scattering force exceeds the gradient force, the stability of the trap decreases, making it more difficult to keep the particle inside the trap. In addition, the thickness of the two triangular silicon elements from tip to tip is also a critical parameter influencing the electromagnetic field distribution and, thus, the stability of the nanocavity.

**Figure 11:**
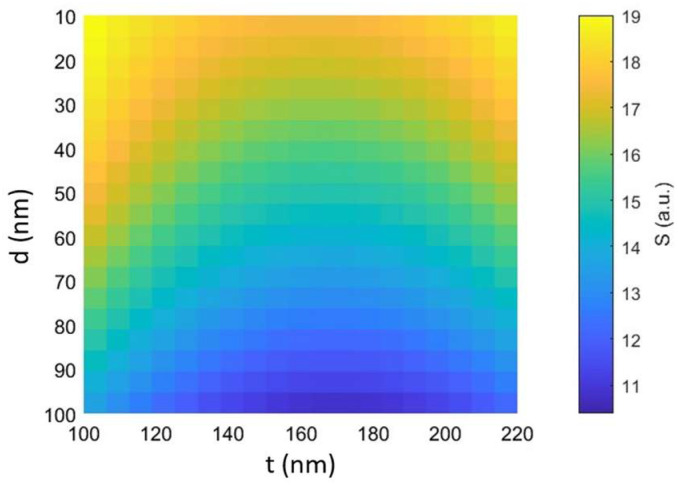
Trapping stability as a function of the particle diameter and the thickness of two dielectric layers for nanobowtie with a silver layer with P_in_ = 10 mW/μm^2^.

In this regard, Figure 11 shows the top view of the 3D graph, illustrating how the stability varies simultaneously as both the particle diameter (d) and the thickness of the dielectric layers (t) change in the case of the structure under study with a silver layer with P_in_ = 10 mW/μm^2^. Figure 11 demonstrates that trapping stability increases as particle diameter decreases, and thinner dielectric layers positively affect trapping stability, albeit to a lesser extent. In particular, thinner dielectric layers tend to concentrate the electromagnetic field more effectively within the cavity, creating regions of high intensity that can marginally increase the gradient strength and improve entrapment stability. These results confirm, however, that particle diameter plays a more significant role in influencing overall trapping stability than silicon layer thickness. For smaller particle diameters, the increase in stability is more pronounced, while changes in dielectric layer thickness have a relatively minor effect.

The choice of a particle diameter of 100 nm and a dielectric thickness of 220 nm for the proposed configuration is justified by considerations of fabrication feasibility and biological characterization, as demonstrated in the literature [15,16,17].

Lastly, we study the trapping behavior of the proposed nanobowtie by considering the trapping stiffness k_i_, expressed as (∂Fi)/∂i with i = x, y, z at the equilibrium point. The trapping stiffness, therefore, is the derivative of the restoring force with respect to the position perturbation around the equilibrium point. When a particle is trapped in an optical trap, the trapping stiffness denotes the force exerted by the trap in response to the particle displacement from its equilibrium position. A significant stiffness value guarantees the stable positioning of the tested particle at the equilibrium position. For instance, an efficient optical trap for polystyrene nanospheres with diameters of 20 nm and 100 nm by using two silicon pillars within a ring resonator was demonstrated in ref. [16], calculating a trapping stiffness of 0.01 fN/(nm/mW) with an input power of 10 mW.

However, the optical tweezers found in the literature, used for trapping 100 nm beads with a power of 10 mW, exhibit high stiffness values around 0.3 fN/nm [7,19,40,42]. In this work, we demonstrate a value of k_z_ = 2.238 fN/nm for the configurations with the silver layer and k_z_ = 2.293 fN/nm for the configurations with the gold layer with P_in_ = 10 mW, calculated over a displacement range from 60 nm to 500 nm.

The proposed device shows a notable improvement in the trap stiffness value compared to other dielectric and plasmonic tweezers reported in the literature. It can be observed that the trapping stiffness increases with the power intensity of the trapping laser. The results obtained confirm the effectiveness of the proposed nanobowtie in trapping nanospheres with a diameter of 100 nm. This size is particularly relevant as it aligns with the typical dimensions of existing viruses, such as the SARS-CoV-2. The demonstrated ability of our device to ensure extremely high trapping stability and stiffness in trapping a particle of these dimensions suggests promising applications in manipulating pathogens, such as viruses, at the nano level.

## 4. Discussion and Conclusions

Our research introduces an innovative dielectric–plasmonic hybrid nanobowtie, serving as a highly efficient nanoparticle trapping device at the nanoscale. The strong light–matter interaction is achieved by incorporating a metal layer beneath the two semiconductor TTSWs. Adding the Au/Ag layer significantly enhances the electromagnetic field density at the trapping site, resulting in increased stability for trapping in the proposed device. This presents an opportunity for achieving superior performance compared to traditional dielectric nanobowties in SOI technology [15,44]. Specifically, we have demonstrated an optical force of 2.9 fN with stability greater than 10 and stiffness exceeding 2.2 fN/nm for trapping a 100 nm bead, which is representative of virus families such as Coronaviridae or Paramyxoviridae, Influenza A, or HIV or more generically small biological objects, for example, nanometric extracellular vesicles sub-groups. The obtained results not only validate the efficacy of the proposed optical nanocavity in trapping a single biological nanoparticle but also showcase a substantial advancement compared to other reported dielectric and/or plasmonic nanobowtie configurations in the literature [9,15,17] both in terms of trapping stability and stiffness, as well as in overcoming thermal challenges. With this configuration, power density values of the order of 10 mW produced exceptionally high trapping stability (S > 10), making optical control of the trap more precise and promoting longer-lasting sample trapping. This feature is particularly attractive for applications requiring high reproducibility and the trapping of biological nanoparticles for extended periods. Increasing the laser power improves the stability trapping number but, at the same time, introduces additional heat to the trap site. The overheating ∆T of the trapping site of approximately 64 K provides the advantage of greater resistance to external perturbations, such as mechanical vibrations. For this reason, this work aims to find a balance to achieve optimum performance in terms of stability and stiffness of the trap while preserving the integrity, vitality, and biological function of the nanoparticle. This must be achieved by maintaining a heating temperature that does not pose a risk to the biological sample. Overall, the use of input optical power to aid or detract from particle trapping stability in optical nanocavity adds another level of control compared to previous experimental devices [16,44] and is a step towards the development of a robust trapping system. Indeed, given the simple geometry, the use of readily available materials, and the ease of fabrication, the possibility of creating an array of the proposed nanobowtie becomes apparent as a potential future development. For instance, such an array could demonstrate effective trapping and manipulating of many nanoparticles in parallel, thereby opening new perspectives in therapeutic and diagnostic applications for various diseases, ranging from infectious diseases to cancer or neurodegenerative disorders. This includes the detection of nano-biological objects like extracellular vehicles (EVs), including microvesicles (MVs) and exosomes, as well as outer membrane vesicles (OMVs) [46].

In conclusion, this research provides theoretical and simulation-based support to advance the trapping of biological particles and to develop future applications like single-molecule fluorescence and/or surface-enhanced Raman spectroscopy (SERS). This enables the investigation, treatment, and monitoring of various diseases, as well as serving as a tool for exploring the effects of new drugs on individual pathogens.

## Figures and Tables

**Table 1:** Summary comparison Table showing the stability of trapping for each input power value and trapped particle size in several previous studies.

	Configuration	Type of Work	Trapping Stability	Size of the Trapped Particle	Input Power Density	Ref.
1.	Dielectric Nanobowtie	Simulated device	1.2	100 nm	6 mW/μm^2^	[15]
2.	Slot Silicon Waveguides	Experimental device	10	75 nm	>250 mW/μm^2^	[45]
3.	Dielectric–Plasmonic Nanobowtie	Simulated device	10.565	100 nm	10 mW/μm^2^	Proposed Au
4.	Dielectric–Plasmonic Nanobowtie	Simulated device	10.370	100 nm	10 mW/μm^2^	Proposed Ag

## Data Availability

Data are contained within the article.
